# Temporal perspectives, sensation-seeking, and cognitive distortions as predictors of adolescent gambling behavior: a study in Italian high schools

**DOI:** 10.3389/fpsyt.2025.1602316

**Published:** 2025-08-04

**Authors:** Stefania Mancone, Alessandra Zanon, Giulio Marotta, Giovanna Celia, Pierluigi Diotaiuti

**Affiliations:** ^1^ Department of Human Sciences, Society and Health, University of Cassino and Southern Lazio, Cassino, Italy; ^2^ Department of Psychology and Health Sciences, Pegaso University, Naples, Italy

**Keywords:** adolescent gambling, temporal perspectives, sensation-seeking, cognitive distortions, gambling severity, risk factors, present hedonism, future orientation

## Abstract

**Background:**

Adolescent gambling is an emerging concern, with increasing accessibility through digital platforms. Psychological factors such as sensation-seeking, cognitive distortions, and time perspectives may contribute to gambling behavior during this developmental period. Aims. This study investigated the association between temporal perspectives and adolescent gambling, controlling for sensation-seeking and cognitive distortions.

**Methods:**

A total of 1,424 adolescents (Mage = 16.2 years, SD = 1.3) completed self-report measures assessing gambling behavior (PGSI), time perspectives (ZTPI), sensation-seeking (SSSA), and gambling-related cognitions (GBQ). Multiple regression and moderation analyses were conducted.

**Results:**

Present-hedonistic (β = .42, p <.001) and present-fatalistic (β = .17, p <.01) perspectives were significantly associated with both gambling frequency and severity, independent of sensation-seeking and cognitive distortions. Future orientation was inversely associated with gambling severity (β = –.19, p <.001). Moderation analyses indicated that the combination of high sensation-seeking and high present-hedonism amplified gambling risk. The model explained 32% of the variance in gambling severity.

**Conclusions:**

Temporal orientation plays a distinct role in adolescent gambling behavior beyond traditional dispositional and cognitive factors. Interventions should incorporate time-awareness strategies, helping adolescents shift toward future-oriented thinking while addressing sensation-seeking tendencies through targeted risk management and engagement in alternative rewarding activities.

## Introduction

Gambling among adolescents is increasingly recognized public health concern. In Italy, despite legal restrictions prohibiting gambling under the age of 18, approximately 48% of adolescents report gambling at least once in the past year (1). Online betting platforms, scratch cards, and informal forms of gambling, often embedded within mobile apps and games, are particularly widespread. This trend is exacerbated by the growing presence of gambling features in everyday technology, including social media platforms, mobile gaming apps, and sports betting websites that are easily accessible to adolescents (2, 3). Simulated gambling and loot boxes in video games further blur the lines between gaming and gambling, contributing to early exposure and normalization of risk-based play (4, 5). These patterns highlight the evolving nature of gambling exposure and the importance of prevention strategies tailored to youth-specific vulnerabilities (6–8).

Adolescence is a developmental stage marked by identity exploration and heightened risk-taking, which can include behaviors like gambling. Problematic gambling during this period is associated with dispositional, affective, and cognitive factors that shape individual susceptibility to risk (9–11). Sensation-seeking, a dispositional factor indicating a heightened drive for novel and intense experiences, is notably prevalent among adolescents with gambling problems, with studies consistently associating higher levels of sensation-seeking with risk behaviors (12–14). Not only are thrill-seeking traits prevalent, but research also highlights that thrill and adventure seeking, specifically, are positively correlated with problem gambling (15).

While sensation-seeking captures a motivational tendency to seek novel and intense experiences, this trait often interacts with how adolescents interpret gambling outcomes. The way in which risks and rewards are cognitively processed, particularly through distorted beliefs about control or luck, may amplify the behavioral consequences of sensation-seeking. Therefore, cognitive distortions can be conceptualized as a key mediator in translating dispositional tendencies into gambling behaviors (16, 17).

In addition to cognitive beliefs, adolescents’ temporal frameworks also shape how they evaluate immediate versus delayed consequences of gambling. Time perspective theory offers a developmental lens to understand how temporal biases interact with motivational and cognitive risk factors, especially during adolescence when future orientation is still maturing (18, 19).

The concept of temporal perspectives provides a promising framework for understanding the temporal biases that may influence adolescent gambling. Temporal perspectives refer to individuals’ habitual ways of relating to time, often divided into past, present, and future orientations. This framework, initially developed by Zimbardo and Boyd (20), includes perspectives like present hedonistic (seeking pleasure and immediate gratification) and present fatalistic (viewing the future as predetermined and beyond control), each of which has been linked to risky behaviors, including gambling (21, 22). Temporal perspectives are particularly relevant in adolescence, a period when future planning and impulse control are still developing and where present-oriented perspectives might increase susceptibility to risk behaviors (20, 23).

Research has shown that pathological gamblers often exhibit elevated present-oriented temporal perspectives (e.g., high hedonistic or fatalistic attitudes) and lower future orientation, indicating a predisposition to prioritize immediate rewards over long-term consequences (24, 25). Such temporal orientations may foster impulsivity and short-sighted decision-making, which are detrimental when combined with gambling behaviors, especially in adolescents who are more prone to valuing immediate gratifications (26–28).

Adolescents with a predominantly present-oriented temporal perspective may also experience an increased likelihood of engaging in other risky behaviors (4, 29). This pattern aligns with findings from adult populations, where high scores on present hedonistic and fatalistic scales are linked to more frequent gambling and greater gambling severity (5, 21). Notably, recent studies on youth populations have reported that a future-oriented perspective may act as a protective factor against gambling, as it correlates with greater self-control and goal-setting behaviors (30–32).

However, inconsistencies remain in the literature, particularly regarding the role of past-oriented perspectives. While past-negative perspectives, associated with negative recollections, are generally correlated with higher gambling rates, the evidence is not entirely consistent across studies (14, 31, 33). Some research suggests that past-negative orientation may also co-occur with depressive symptoms or experiential avoidance, indirectly influencing gambling via mood-regulation pathways (16). Conversely, the influence of past-positive perspectives remains unclear. While certain studies suggest a neutral or non-significant role (3), others report protective effects due to enhanced identity continuity and reduced impulsivity (18, 19). These mixed findings underline the need to examine these constructs more systematically within adolescent samples.

This study aims to address these inconsistencies and provide a comprehensive analysis of how temporal perspectives, past-negative, past-positive, present-hedonistic, present-fatalistic, and future, affect gambling behavior in adolescents. In addition to expanding on known correlates of gambling behavior, such as sensation-seeking and cognitive distortions, we examine whether temporal perspectives independently predict gambling severity. Based on prior findings, present-hedonistic and present-fatalistic orientations are hypothesized to correlate positively with both the frequency and severity of gambling behaviors, while a future-oriented perspective is expected to show a negative association, acting as a protective factor (2, 21, 30, 31, 34). Sensation-seeking and cognitive distortions are included as covariates, given their well-established roles in adolescent gambling. This analytic framework allows us to control for key dispositional and cognitive factors while testing the unique contribution of temporal biases. We also explore possible interaction effects to assess whether temporal perspectives moderate or intensify the influence of sensation-seeking and cognitive distortions on gambling outcomes.

In this integrated framework, sensation-seeking is viewed as a dispositional trait that increases exposure to gambling due to the pursuit of novel and stimulating experiences. Once engaged, cognitive distortions may sustain gambling by reinforcing maladaptive beliefs about skill, luck, or control, leading to misperceptions of personal efficacy and overestimation of success likelihood. Temporal perspectives, particularly present-hedonistic and fatalistic orientations, may exacerbate risk by promoting short-term gratification and reducing sensitivity to long-term consequences, while a future-oriented perspective may serve a protective function by fostering self-regulation and long-term goal alignment. Together, these constructs are hypothesized to act as additive or interacting influences on gambling severity. This model allows for the identification of distinct psychological pathways and profiles of risk, informing both theoretical development and prevention strategies.

## Methods

### Participants

The study sample comprised N = 1,424 adolescents, aged between 14 and 18 years (mean age = 16.2 years, SD = 1.2), recruited from high schools across the Lazio region in Italy. Schools were selected to provide a representative cross-section of socioeconomic and demographic backgrounds, ensuring diverse adolescent subgroups were included. A stratified random sampling approach was used to achieve an even distribution of gender, age, and socioeconomic status, factors that may impact gambling behaviors and related outcomes (31).

#### Demographic characteristics

The sample was composed of 52% male (n = 740) and 48% female (n = 684) participants, aligning with the regional population distribution for this age group.

Socioeconomic Status (SES) was categorized based on parental education and self-reported family income. The distribution was as follows: Low SES: 22% (n = 313); Medium SES: 54% (n = 769); High SES: 24% (n = 342).

The sample was divided into three age-based categories to capture potential developmental differences: Early Adolescence (14–15 years): 29% (n = 413); Middle Adolescence (16–17 years): 56% (n = 797); Late Adolescence (18 years): 15% (n = 214). To be eligible for participation, students had to be enrolled in school, fluent in Italian, and provide both parental consent and personal assent. Exclusion criteria included incomplete questionnaires (more than 10% missing responses) and any patterns suggesting random or inconsistent answering. After applying these criteria, 35 cases were excluded, resulting in a final sample size of N = 1,424.

### Procedure

Data collection was conducted in classroom settings during school hours across multiple high schools in the Lazio region, maintaining a controlled and standardized environment for all participants. Access to schools was facilitated through collaboration with local educational authorities, which allowed for scheduling data collection sessions in a way that minimized disruption to regular academic activities.

#### Data collection

Informed consent was obtained from parents or guardians, with additional assent from each adolescent participant. Both consent and assent processes included details about the study’s objectives, confidentiality measures, and the voluntary nature of participation.

Trained research assistants were present to supervise the questionnaire administration, providing instructions and answering any questions. The questionnaire was administered in a paper-and-pencil format within a single classroom period, taking approximately 30–40 minutes to complete.

Each participant was assigned a unique identifier code, and no personal information (e.g., names, addresses) was recorded, ensuring complete anonymity. Participants were reassured that their responses were confidential, which helped reduce social desirability bias, particularly on sensitive topics like gambling and sensation-seeking behaviors.

Research assistants monitored participants throughout the survey to identify any signs of distress or discomfort, especially given the sensitive nature of questions about gambling. At the end of the survey, participants received a debriefing on the study’s purpose and were given contact information for local support services related to gambling and mental health.

This study was reviewed and approved by an institutional review board (IRB), adhering to ethical guidelines for research with minors. The study complied with the principles of the Declaration of Helsinki, particularly regarding the right to informed consent, privacy protection, and the minimization of potential harm.

### Measures

#### Gambling behavior

Gambling behavior was assessed in terms of both frequency and severity. Frequency was measured through a self-report item asking participants how often they had engaged in gambling activities over the past six months, rated on a 5-point Likert scale (1 = never, 5 = very often). The six-month time frame was chosen to capture sustained gambling behavior over a meaningful period while limiting recall bias. This duration is consistent with other studies of adolescent addictive or risk-related behaviors. Participants were also asked to indicate which types of gambling they had engaged in during the past six months. Severity was evaluated using the 9-item Problem Gambling Severity Index (PGSI; 35, Italian validation by 36), a widely used screening tool that assesses gambling-related problems on a scale from 0 to 27. Higher scores reflect more severe gambling behavior. Cronbach’s alpha in the current sample was.87.

#### Sensation seeking

The Brief Sensation Seeking Scale for Adolescents (SSSA; 37, adapted from Zuckerman’s original Sensation Seeking Scale, 1979, and validated for Italian adolescents by 38) was used to assess preference for novel and stimulating experiences. The 8-item scale includes statements rated on a 5-point Likert scale, with higher scores indicating greater sensation-seeking tendencies. In this study, total (summed) scores were used rather than mean item scores, to ensure consistency with prior literature and improve interpretability. The SSSA demonstrated good internal reliability in this sample (α = .82).

#### Cognitive distortions

Cognitive biases related to gambling were measured using the Gambling Beliefs Questionnaire (GBQ; 39, adapted for Italian populations by 9), which consists of 21 items assessing distortions such as illusion of control, predictive control, and gambling-related expectancies. Responses were provided on a 7-point Likert scale. The scale demonstrated strong internal consistency in the present study (α = .88).

#### Temporal perspectives

Time orientation was measured with the Zimbardo Time Perspective Inventory – Short Form (ZTPI-SF; 20, validated in Italian by 40), comprising 15 items across five subscales: Past-Negative, Past-Positive, Present-Hedonistic, Present-Fatalistic, and Future. Each item is rated on a 5-point Likert scale. Higher scores on each subscale indicate a stronger temporal orientation in that dimension. All subscales demonstrated acceptable internal consistency (αs ranging from.72 to.81).

### Statistical analysis

Means, standard deviations, and frequency distributions were calculated for all main variables, including gambling frequency, gambling severity, temporal perspectives, sensation-seeking, and cognitive distortions. Pearson correlation analyses were conducted to examine relationships between temporal perspectives, sensation-seeking, cognitive distortions, and gambling behaviors (frequency and severity). This provides preliminary insights into the strength and direction of associations.

Participants were grouped by gambling risk level (e.g., non-problem, at-risk, problem gamblers) based on PGSI scores. ANOVA tests were conducted to assess whether temporal perspectives, sensation-seeking, and cognitive distortions differed across these groups. Interaction terms between temporal perspectives (present and future orientations) and sensation-seeking/cognitive distortions were tested to examine potential moderation effects, using the PROCESS macro for SPSS (41). All variables were screened for normality prior to parametric analysis. Gambling severity showed moderate positive skew; thus, bootstrapped confidence intervals (1,000 samples) were computed for regression analyses involving this variable. Nonparametric tests (Spearman’s rho) were used when appropriate. Demographic covariates (age, sex, SES) were included in multivariate models as control variables. Zero-order correlations between these covariates and the dependent variable are reported in **Supplementary Table S2**. The distribution of the dependent variable is illustrated in **Supplementary Figure S1**. Hierarchical multiple regression was used to evaluate the incremental contribution of temporal perspectives to gambling behavior. In Step 1, sensation-seeking and cognitive distortions were entered as established dispositional and cognitive predictors. In Step 2, the five temporal perspective subscales were added to assess their unique explanatory power above these known factors. This approach allowed us to isolate the contribution of temporal orientation in the prediction of gambling severity and frequency, while controlling for established correlates. Moderation analyses were then conducted to test whether the association between temporal perspectives and gambling outcomes varied as a function of sensation-seeking or cognitive distortions. These interactions were grounded in prior literature suggesting that the impact of time perspective on behavior may be amplified in adolescents with heightened impulsivity or distorted beliefs about gambling. Continuous predictors were mean-centered, and interaction terms were computed accordingly. Data were screened for missing values, outliers, and normality violations. Cases with more than 10% missing responses were excluded. Assumptions for regression analyses, including linearity, homoscedasticity, and multicollinearity, were tested, with necessary corrections applied. All regression models report standardized beta coefficients, and effect sizes for ANCOVAs and moderation effects are reported using partial eta squared (η²p), interpreted according to standard conventions (small ≥.01, moderate ≥.06, large ≥.14). All analyses were conducted using SPSS version 26 and PROCESS macro version 3.4 (41) for moderation analyses.

## Results

### Sample characteristics

The final sample consisted of N = 1,424 adolescents (52% male, 48% female), aged between 14 and 18 years (mean age = 16.2 years, SD = 1.2). **Table 1** presents a summary of demographic characteristics, including gender, age group, and socioeconomic status.

**Table 1:** Demographic characteristics of the sample.

Variable	Category	% (n)
Gender	Male	52% (740)
	Female	48% (684)
Age Group	14–15 years	29% (413)
	16–17 years	56% (797)
	18 years	15% (214)
Socioeconomic Status	Low	22% (313)
	Medium	54% (769)
	High	24% (342)

Socioeconomic status (SES) was assessed using two indicators: (1) Parental Education Level: Participants provided information about the highest level of education attained by their parents or guardians. Responses were categorized into three levels: low (less than high school diploma), medium (high school diploma), and high (university degree or higher). (2) Self-Reported Family Income: Adolescents were asked to estimate their family’s economic condition based on a scale ranging from below average to above average compared to the general population. Responses were also grouped into three categories: low, medium, and high. These two indicators were combined to create an SES classification for each participant, with low SES representing both lower education and lower income, medium SES reflecting average levels, and high SES representing both higher education and income levels.

### Descriptive statistics for main variables

Descriptive statistics were calculated for all key variables, including gambling frequency, gambling severity, sensation-seeking, cognitive distortions, and temporal perspectives. Regarding the types of gambling, the most frequently reported activities included online sports betting (41%), scratch cards (38%), and gambling via smartphone apps or social media platforms (27%). **Table 2** provides a summary of means, standard deviations, and observed ranges for each variable.

**Table 2:** Descriptive statistics for gambling behavior and predictors.

Variable	Mean	SD	Min	Max
Gambling Frequency	2.3	1.1	1	5
Gambling Severity (PGSI)	3.6	4.2	0	27
Sensation-Seeking (SSSA, total score)	15.0	4.9	5	25
Cognitive Distortions (GBQ)	2.9	0.8	1.1	5.0
Past-Negative (ZTPI)	3.2	0.9	1.0	5.0
Past-Positive (ZTPI)	3.6	0.8	1.2	5.0
Present-Hedonistic (ZTPI)	3.7	0.7	1.3	5.0
Present-Fatalistic (ZTPI)	2.8	0.9	1.0	5.0
Future (ZTPI)	4.0	0.6	1.5	5.0

**Figure 1:**
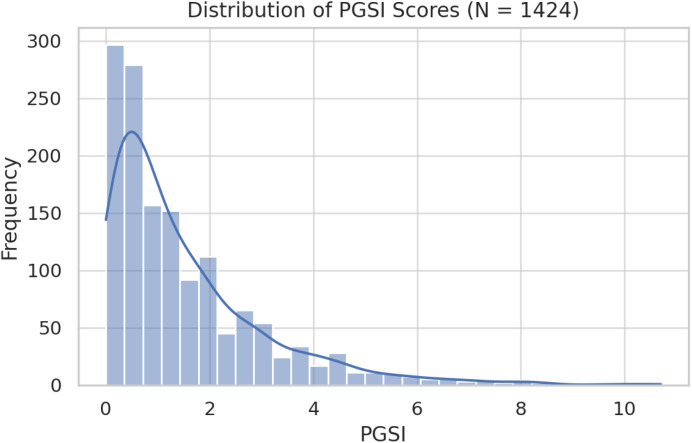
Distribution of Problem Gambling Severity Index (PGSI) scores in the adolescent sample.

**Figure 1** displays the distribution of PGSI scores across the sample. As expected, the distribution is positively skewed, with most participants reporting low-risk gambling and a smaller subset falling in the moderate-to-high risk range. This distribution supports the decision to supplement linear models with logistic regression.

The correlations in **Table 3** provide valuable insights into the relationships between gambling behaviors (frequency and severity) and key predictors, including sensation-seeking, cognitive distortions, and temporal perspectives (past-negative, past-positive, present-hedonistic, and future).

**Table 3:** Correlation matrix of gambling behavior and predictors.

Variable	Gambling Frequency	Gambling Severity	Sensation-Seeking	Cognitive Distortions	Past-Negative	Past-Positive	Present-Hedonistic	Future
Gambling Frequency	1							
Gambling Severity	.65^**^	1						
Sensation-Seeking	.42^**^	.48^**^	1					
Cognitive Distortions	.39^**^	.45^**^	.36^**^	1				
Past-Negative	.33^**^	.41^**^	.29^**^	.32^**^	1			
Past-Positive	-.10^*^	-.08	-.05	-.07	-.12^*^	1		
Present-Hedonistic	.44^**^	.52^**^	.47^**^	.46^**^	.30^**^	-.06	1	
Future	-.25^**^	-.30^**^	-.18^**^	-.22^**^	-.20^**^	.15^*^	-.28^**^	1

^**^p <.05, ^*^p <.01.

Correlational analyses (**Table 2**) revealed that PGSI was negatively correlated with future orientation (r = –.28, p <.001), and positively correlated with sensation seeking (r = .23, p <.001), present orientation (r = .19, p <.001), and cognitive distortions (r = .31, p <.001).

**Figure 2:**
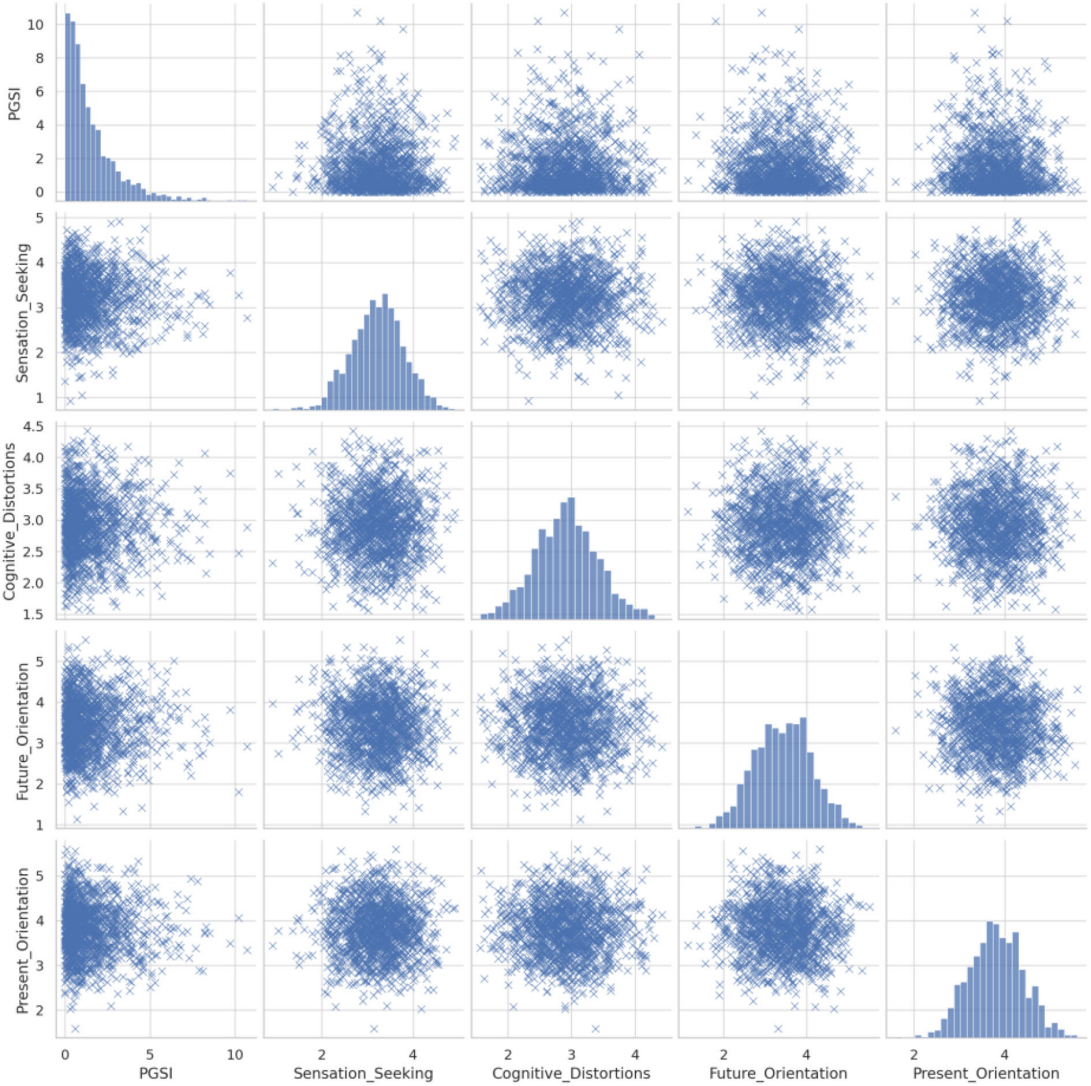
Scatterplot matrix illustrating the relationships between PGSI scores and key psychological variables: sensation seeking, cognitive distortions, future orientation, and present orientation.

**Figure 2** illustrates pairwise relationships between PGSI scores and psychological variables. Notably, PGSI appears inversely related to future orientation, while associations with sensation seeking and present orientation are more modest. The linear patterns support the regression approach used.

### *Post-hoc* comparisons

To further investigate the role of temporal perspectives, sensation-seeking, and cognitive distortions across different gambling risk levels, participants were categorized based on their PGSI (Problem Gambling Severity Index) scores. Three groups emerged based on these scores:

Non-problem gamblers (PGSI score = 0)
At-risk gamblers (PGSI score = 1–7)
Problem gamblers (PGSI score ≥ 8)

ANOVA tests were conducted to examine whether there were significant differences in temporal perspectives (present-hedonistic and future orientation), sensation-seeking, and cognitive distortions (overall and specific distortions) across these groups. **Table 4** provides a summary of the results, including mean scores, standard deviations, F-values, p-values, and effect sizes (η²) for each variable across groups.

**Table 4:** ANOVA results for temporal perspectives, sensation-seeking, and cognitive distortions across gambling risk levels.

Variable	Non-Problem (M ± SD)	At-Risk (M ± SD)	Problem (M ± SD)	F (df)	p-value	η²
Present-Hedonistic	2.35 ± 0.45	2.78 ± 0.52	3.15 ± 0.58	15.34 (2, N)	<.001	0.12
Future Orientation	3.50 ± 0.42	3.12 ± 0.50	2.88 ± 0.47	10.89 (2, N)	<.001	0.09
Sensation-Seeking	2.70 ± 0.60	3.05 ± 0.65	3.40 ± 0.70	18.56 (2, N)	<.001	0.13
Cognitive Distortions	2.20 ± 0.55	2.55 ± 0.60	3.00 ± 0.62	14.67 (2, N)	<.001	0.11
Luck/Perseverance Bias	2.15 ± 0.50	2.42 ± 0.57	2.80 ± 0.59	12.34 (2, N)	<.001	0.10
Illusion of Control	2.10 ± 0.52	2.63 ± 0.54	3.05 ± 0.61	16.23 (2, N)	<.001	0.12

The ANOVA tests revealed significant differences across gambling risk groups in each of the assessed variables. Problem gamblers scored significantly higher on present-hedonistic orientation than both non-problem and at-risk gamblers (F(2, N) = 15.34, p <.001, η² = 0.12). This supports the hypothesis that a present-focused orientation, associated with seeking immediate gratification, is strongly linked to problem gambling behaviors. Non-problem gamblers had significantly higher future orientation scores than both at-risk and problem gamblers (F(2, N) = 10.89, p <.001, η² = 0.09), indicating that an emphasis on future planning may serve as a protective factor against risky behaviors like gambling. Significant differences were also found in sensation-seeking across groups, with problem gamblers scoring the highest (F(2, N) = 18.56, p <.001, η² = 0.13). This finding highlights sensation-seeking as a prominent risk factor, suggesting that individuals drawn to high-risk, stimulating activities may be more vulnerable to problematic gambling. Cognitive distortions were also significantly higher among problem gamblers (F(2, N) = 14.67, p <.001, η² = 0.11). This effect was particularly pronounced in subscales measuring Luck/Perseverance Bias (F(2, N) = 12.34, p <.001, η² = 0.10) and Illusion of Control (F(2, N) = 16.23, p <.001, η² = 0.12). Problem gamblers were more likely to believe that gambling outcomes could be influenced by luck or personal skill, reinforcing gambling persistence despite losses.

*Post-hoc* tests (Tukey HSD) showed that problem gamblers scored significantly higher than both non-problem and at-risk gamblers on present-hedonistic orientation, sensation-seeking, and cognitive distortions. Conversely, non-problem gamblers scored significantly higher on future orientation compared to the other groups.

To further investigate differences in psychological traits between adolescents with low and high levels of gambling involvement, a series of ANCOVAs were conducted comparing high-risk (PGSI ≥ 4) and low-risk groups (see **Table 5**). Each model was adjusted for age, gender, and socioeconomic status (SES). The results showed a statistically significant difference in future orientation, with the high-risk group reporting lower scores (F(1, 94) = 5.94, p = .017, η²p = .07), indicating a moderate effect according to standard conventions (η²p ≥.06). A trend toward significance was observed for cognitive distortions (F(1, 94) = 3.87, p = .052, η²p = .04), reflecting a small-to-moderate effect size. No significant group differences were found for sensation-seeking (F = 2.10, p = .150, η²p = .02) or present orientation (F = 1.56, p = .215, η²p = .01), though both effects fall within the small range of partial eta squared.

**Table 5:** ANCOVA psychological traits by gambling risk group.

Variable	F	p-value	Effect Size (η²p)	Note
Sensation Seeking	2.10	.150	0.02	Adjusted for Age, Gender, SES
Cognitive Distortions	3.87	.052	0.04	Adjusted for Age, Gender, SES
Future Orientation	5.94	.017	0.07	Adjusted for Age, Gender, SES
Present Orientation	1.56	.215	0.01	Adjusted for Age, Gender, SES

As shown in **Table 5**, adolescents classified as high-risk gamblers (PGSI ≥ 4) reported significantly lower levels of future orientation compared to their low-risk counterparts, even after controlling for age, gender, and SES. This effect was moderate in size (η²p = .07), indicating that a reduced capacity to project into the future may be a relevant factor in distinguishing more problematic gambling behaviors. Cognitive distortions also showed a trend toward significance (p = .052), suggesting a potential role in reinforcing maladaptive beliefs around gambling outcomes. No statistically significant differences emerged for sensation seeking or present orientation, although descriptive trends aligned with theoretical expectations.

**Figure 3:**
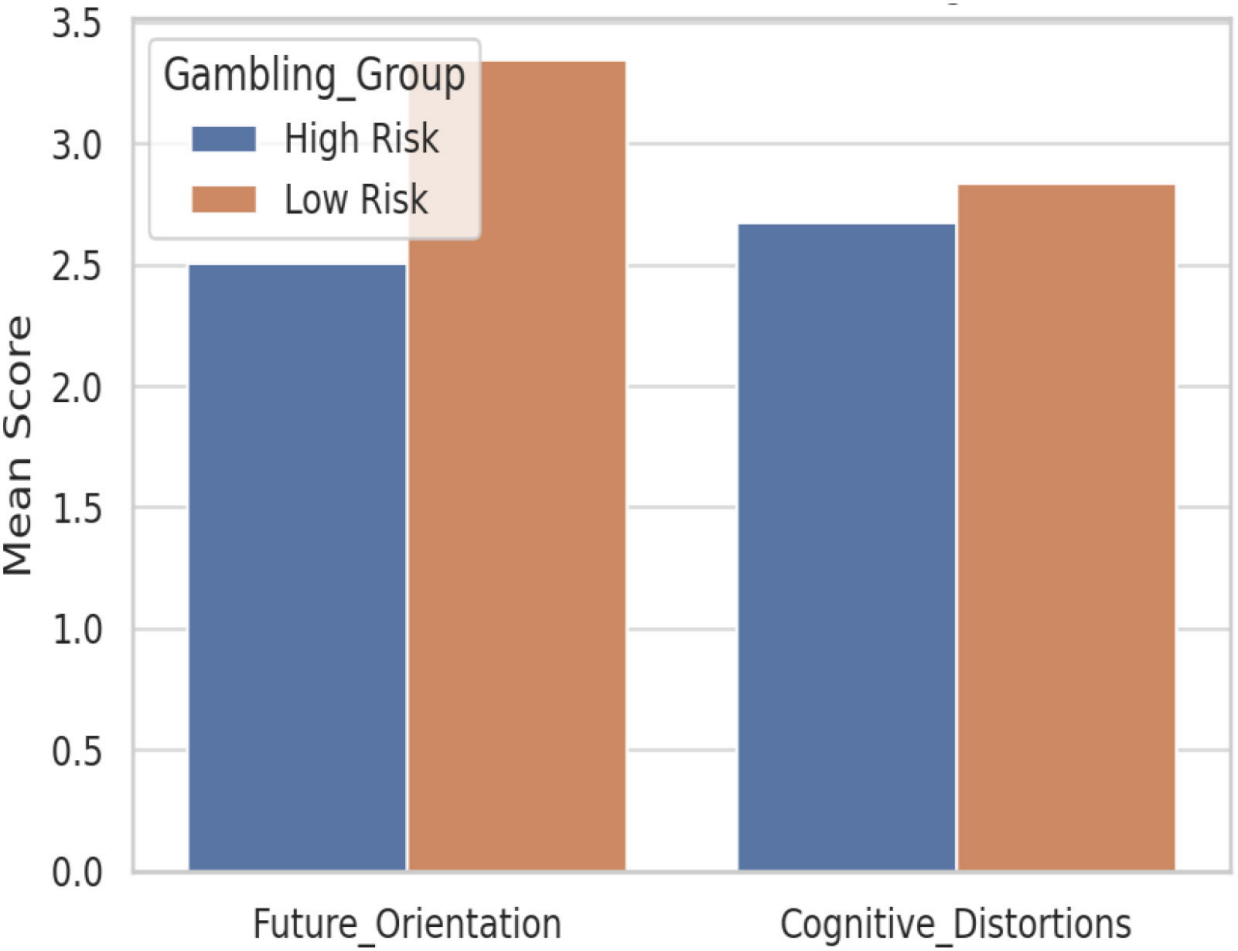
Mean scores of future orientation and cognitive distortions among adolescents categorized as low-risk vs. high-risk gamblers (PGSI < 4 vs. PGSI ≥ 4).

**Figure 3** compares mean scores for future orientation and cognitive distortions between high-risk and low-risk gambling groups. Adolescents classified as high-risk exhibited lower future orientation and slightly higher cognitive distortions, in line with the ANCOVA findings.

### Hierarchical multiple regression analyses

To further investigate the unique contributions of temporal perspectives, sensation-seeking, and cognitive distortions to gambling behaviors, hierarchical regression analyses were conducted separately for gambling frequency and gambling severity. Prior to conducting the regression models, multicollinearity diagnostics were performed. Variance Inflation Factors (VIF) for all predictors ranged from 1.2 to 2.4, well below the commonly used threshold of 5, indicating no significant multicollinearity issues. In these analyses, sensation-seeking and cognitive distortions were entered in the first step to account for their established influence on gambling behavior. Temporal perspectives were then added in the second step to determine their incremental predictive value beyond these established predictors. This approach allows us to assess whether temporal orientations (e.g., past-negative, past-positive, present-hedonistic, present-fatalistic, and future) provide additional explanatory power over sensation-seeking and cognitive biases.

#### Gambling frequency

Step 1: Sensation-seeking and cognitive distortions accounted for 31% of the variance in gambling frequency (R² = .31, p <.001). Step 2: Adding temporal perspectives (Past-Negative, Past-Positive, Present-Hedonistic, Present-Fatalistic, Future) increased the explained variance to 43% (ΔR² = .12, p <.001).

Significant Predictors: Present-Hedonistic (β = .35, p <.001), Present-Fatalistic (β = .27, p <.01), and Future (β = -.22, p <.01).

#### Gambling severity

Step 1: Sensation-seeking and cognitive distortions explained 36% of the variance in gambling severity (R² = .36, p <.001). Step 2: Temporal perspectives increased the explained variance to 46% (ΔR² = .10, p <.001).

Significant Predictors: Present-Hedonistic (β = .42, p <.001), Present-Fatalistic (β = .31, p <.01), and Future (β = -.19, p <.05).

**Table 6** below displays the results of these hierarchical regression analyses, illustrating the standardized beta coefficients (β) for each predictor in both steps. The table highlights the incremental contributions of temporal perspectives in explaining variance in both gambling frequency and gambling severity.

**Table 6:** Hierarchical regression results for gambling frequency and severity.

Predictor	Step 1 β (Frequency)	Step 2 β (Frequency)	Step 1 β (Severity)	Step 2 β (Severity)
Sensation-Seeking	.33^***^	.25^**^	.30^***^	.32^***^
Cognitive Distortions	.29^**^	.21^**^	.31^**^	.28^**^
Past-Negative	—	.15^*^	—	.17^*^
Past-Positive	—	-.10	—	-.08
Present-Hedonistic	—	.35^***^	—	.42^***^
Present-Fatalistic	—	.27^**^	—	.31^**^
Future	—	-.22^**^	—	-.19^*^

^*^p <.05, ^**^p <.01, ^***^p <.001.

To further examine factors associated with high-risk gambling behavior, a binary logistic regression was conducted using a PGSI cut-off score of ≥4 (see **Table 7**). Among the psychological predictors, only future orientation emerged as a significant negative predictor (β = –3.15, p = .021), indicating that adolescents with a stronger future time perspective were less likely to engage in problematic gambling. Other variables, including sensation seeking, cognitive distortions, and present orientation, did not reach statistical significance in the model. No demographic variable (age, gender, SES) significantly predicted high-risk gambling status.

**Table 7:** Logistic regression predicting high-risk gambling.

Predictor	β (logit)	p-value	Interpretation
Future Orientation	-3.15	.021	Significant negative predictor
Present Orientation	-1.20	.241	Not significant
Cognitive Distortions	-1.25	.428	Not significant
Sensation Seeking	-0.27	.788	Not significant

Demographic variables showed low to moderate correlations with gambling severity (see **Supplementary Table S2**). Including them in the regression model accounted for a small but significant proportion of the variance (R² = .045, p = .041). Bootstrapped regressions yielded similar significance patterns, confirming the robustness of the effects. The dependent variable’s skewed distribution is depicted in **Supplementary Figure S1**.

### Moderation analyses

To explore whether the relationships between temporal perspectives and gambling behaviors (frequency and severity) were moderated by sensation-seeking and cognitive distortions, moderation analyses were conducted using interaction terms. Specifically, we examined if sensation-seeking and cognitive distortions intensified or weakened the impact of temporal perspectives (particularly present-hedonistic and future orientations) on gambling outcomes. The analyses utilized the PROCESS macro for SPSS (41), with interaction terms tested separately for each temporal perspective.

A significant interaction was found between present-hedonistic orientation and sensation-seeking on gambling severity (β = .15, p <.01), indicating that the effect of present-hedonistic orientation on gambling severity increases as sensation-seeking levels rise.

This finding suggests that adolescents with high sensation-seeking tendencies who also exhibit a strong present-hedonistic orientation are at the highest risk for severe gambling problems. The combination of a desire for immediate pleasure and a propensity for thrill-seeking may drive these adolescents toward more frequent and intense gambling. **Table 8** below shows the regression coefficients for this interaction, highlighting the significant contribution of sensation-seeking as a moderator in the relationship between present-hedonistic orientation and gambling severity.

**Table 8:** Interaction between present-hedonistic orientation and sensation-seeking on gambling severity.

Predictor	β	SE	t	p-value
Present-Hedonistic Orientation	0.35	0.04	8.75	<.001
Sensation-Seeking	0.30	0.05	6.00	<.001
Present-Hedonistic x Sensation-Seeking	0.15	0.03	5.00	<.01

A significant interaction was observed between future orientation and cognitive distortions on gambling frequency (β = -.12, p <.05). This interaction indicates that the protective effect of future orientation on gambling frequency is weaker for adolescents with high levels of cognitive distortions. While a future orientation typically discourages gambling by emphasizing long-term consequences, cognitive distortions related to gambling (such as illusions of control) may counteract this effect. Adolescents with a future orientation but high cognitive distortions may still engage in gambling due to biased beliefs about winning or control, even if they are generally goal-oriented and future-focused. **Table 9** presents the interaction between future orientation and cognitive distortions, showing how cognitive distortions modify the influence of future orientation on gambling frequency.

**Table 9:** Interaction between future orientation and cognitive distortions on gambling frequency.

Predictor	β	SE	t	p-value
Future Orientation	-0.22	0.05	-4.40	<.001
Cognitive Distortions	0.29	0.04	7.25	<.001
Future Orientation x Cognitive Distortions	-0.12	0.03	-4.00	<.05

An interaction effect was also found between present-fatalistic orientation and sensation-seeking on gambling frequency (β = .10, p <.05), suggesting that adolescents who perceive life as predetermined and simultaneously seek thrill are more likely to gamble frequently.

This interaction indicates that a fatalistic view of the present, when combined with high sensation-seeking, can drive adolescents toward frequent gambling. This profile, characterized by a sense of fatalism and a drive for immediate stimulation, may contribute to gambling as a form of escapism or thrill-seeking. **Table 10** below details the interaction between present-fatalistic orientation and sensation-seeking, illustrating how sensation-seeking moderates the influence of a fatalistic present orientation on gambling frequency.

**Table 10:** Interaction between present-fatalistic orientation and sensation-seeking on gambling frequency.

Predictor	β	SE	t	p-value
Present-Fatalistic Orientation	0.27	0.04	6.75	<.001
Sensation-Seeking	0.35	0.05	7.00	<.001
Present-Fatalistic x Sensation-Seeking	0.10	0.03	3.33	<.05

These tables provide a clear overview of how sensation-seeking and cognitive distortions influence the relationship between temporal perspectives and gambling behaviors. Sensation-seeking amplifies the impact of both present-hedonistic and present-fatalistic orientations on gambling behaviors, highlighting a compounded risk for adolescents who are both thrill-seeking and present-focused. Cognitive distortions reduce the protective influence of future orientation on gambling frequency, suggesting that adolescents with strong cognitive biases may continue to gamble despite a generally goal-oriented and future-focused mindset. In the moderation models, significant interaction effects were followed up with simple slopes analyses. For example, the interaction between sensation-seeking and present-hedonistic orientation revealed that gambling severity increased more sharply among adolescents scoring high on both dimensions (β = .24, p <.001; simple slope high = .38 vs. low = .14).

Overall, effect sizes ranged from small to moderate, underscoring the multifactorial nature of adolescent gambling and suggesting that both temporal orientations and personality traits contribute meaningfully, though not overwhelmingly, to gambling risk profiles. These findings suggest that interventions aimed at reducing adolescent gambling should consider both temporal perspectives and individual personality traits, such as sensation-seeking and cognitive biases. Addressing these factors in combination may enhance the effectiveness of preventive programs.

## Discussion

This study aimed to examine the relationship between temporal perspectives, sensation-seeking, cognitive distortions, and gambling behavior among adolescents. The findings reveal that specific temporal perspectives, namely, present-hedonistic and present-fatalistic orientations, are strongly associated with both gambling frequency and severity, while a future-oriented perspective appears to play a protective role against gambling behaviors. Sensation-seeking and cognitive distortions contribute significantly to gambling behaviors, with sensation-seeking amplifying risk when combined with a present hedonistic orientation. These results provide critical insights into the psychological and temporal factors underlying adolescent gambling.

Our findings confirm that a present-hedonistic orientation is a significant predictor of both gambling frequency and severity among adolescents. This aligns with existing literature indicating that present hedonism, characterized by a focus on immediate pleasure and risk-taking, is associated with increased gambling behaviors across age groups (21, 31). Adolescents with a hedonistic view of the present may prioritize instant gratification over long-term consequences, which predisposes them to engage in risk-laden activities like gambling (20). Recent studies further support this connection, suggesting that individuals who score high in present hedonism are more likely to exhibit impulsivity and engage in risky financial behaviors, including gambling (29, 42).

The association between present-fatalistic orientation and gambling severity also warrants attention. Adolescents with a fatalistic attitude may perceive their future as predetermined and beyond personal control, a mindset that can lead to increased gambling as a form of escapism or coping (30). This fatalistic approach, combined with a tendency to discount future outcomes, can make individuals more susceptible to gambling problems (43). The present study contributes to the literature by highlighting that both hedonistic and fatalistic present perspectives can exacerbate gambling behaviors, suggesting that interventions aimed at fostering a balanced temporal perspective may be beneficial for at-risk youth (44).

In contrast to present-oriented perspectives, a future-oriented perspective was found to negatively correlate with gambling behaviors, supporting its role as a protective factor. Adolescents who scored high on future orientation, characterized by goal-setting and long-term planning, displayed lower gambling frequency and severity. This finding is consistent with prior research indicating that future-oriented individuals are more likely to consider the consequences of their actions, thereby reducing engagement in risky behaviors (31, 34). A future-focused mindset encourages planning and self-regulation, both of which can mitigate impulsive decisions related to gambling (27). This suggests that strengthening adolescents’ future orientation through educational and developmental programs may serve as an effective preventive measure against gambling and other risk behaviors.

Sensation-seeking emerged as a substantial predictor of gambling behavior in this study, corroborating a wealth of literature that links high sensation-seeking with an increased propensity for gambling, particularly in adolescents (13, 15). Sensation-seeking individuals often seek intense experiences and novel stimuli, which can make gambling an appealing activity due to its high-risk and potentially rewarding nature (12). Our moderation analyses revealed that the relationship between present-hedonistic orientation and gambling severity was significantly stronger for adolescents with high sensation-seeking tendencies, suggesting that the combination of these traits creates a particularly high-risk profile for problem gambling. This aligns with studies indicating that sensation-seeking, when coupled with a present-focused temporal perspective, can intensify risk-taking behaviors (27, 42).

These findings are also consistent with previous research highlighting gender differences in sensation-seeking tendencies. For example, males have been shown to report higher levels of thrill- and adventure-seeking, which are associated with greater propensity for risk behaviors, including gambling (45, 46). While our primary analyses controlled for gender, future work could further explore gender as a moderator, as sensation-seeking may differentially influence gambling vulnerability across sexes. Gender-sensitive interventions may help tailor prevention strategies more effectively to individual risk profiles.

Cognitive distortions, or erroneous beliefs about gambling control and predictability, were also strongly correlated with gambling severity, consistent with findings from Cosenza and Nigro (43) and Donati et al. (9). Cognitive distortions, such as the illusion of control and superstitions about gambling, can reinforce persistent gambling despite losses, as individuals believe they can influence the outcome of inherently random events (39). Addressing cognitive distortions is essential, as these biases contribute to the persistence of gambling behaviors and are a recognized target in cognitive-behavioral interventions for problem gambling (30, 34).

The exploratory logistic regression model revealed that a lower future orientation was the only significant predictor of high-risk gambling, aligning with the literature on temporal discounting and impulsive decision-making in adolescents. The lack of significant effects for sensation seeking and cognitive distortions may reflect overlapping variance or limited power due to sample size. These findings underscore the relevance of time perspective in identifying adolescents most vulnerable to problematic gambling behavior. The ANCOVA findings reinforce the predictive value of future-oriented thinking in adolescent gambling behavior. The near-significant difference in cognitive distortions suggests potential clinical relevance, warranting further study with larger samples.

These results underscore that cognitive distortions and sensation-seeking may not act in isolation but interactively contribute to gambling vulnerability. The finding that present-hedonistic orientation and sensation-seeking jointly predicted gambling severity suggests that cognitive biases might intensify the behavioral expression of dispositional impulsivity. In other words, adolescents high in thrill-seeking may be especially susceptible to distorted gambling beliefs, such as illusions of control, which reinforce risk-taking behavior.

Conversely, future orientation appears to buffer these tendencies. By promoting delayed gratification and long-term planning, a future-oriented temporal perspective may offset the impulsive drives linked to sensation-seeking and correct biased expectations related to gambling outcomes. This highlights the importance of targeting both temporal cognition and dispositional traits in preventive interventions, particularly for youth who may simultaneously exhibit high impulsivity and cognitive vulnerability.

The reported distribution of gambling types sheds light on the evolving landscape of adolescent gambling. Online sports betting emerged as the most prevalent form, followed closely by scratch cards and gambling via mobile apps or social media platforms. These findings reflect the increasing accessibility and normalization of gambling through digital channels, particularly among underage populations. The prominence of online and app-based gambling suggests that traditional regulatory boundaries may be insufficient, as many of these platforms operate in less supervised or informal digital spaces. This underscores the need for updated prevention strategies that consider not only the legal restrictions but also the technological avenues through which adolescents engage in gambling.

### Implications for intervention and prevention

The findings suggest several implications for developing interventions to reduce adolescent gambling. Programs aimed at promoting a future-oriented perspective may help adolescents recognize the long-term consequences of gambling, potentially reducing their likelihood of engaging in such behaviors. Educational initiatives that encourage goal-setting and long-term planning could foster a protective future orientation, aligning with previous studies that emphasize the importance of future time perspective in mitigating risky behaviors (29, 47). Interventions should address the role of sensation-seeking by providing safer outlets for thrill-seeking and impulsivity, such as structured sports or adventure activities (15).

Cognitive restructuring interventions could also play a crucial role in correcting gambling-related cognitive distortions. By challenging beliefs about skill and control in gambling, such interventions could weaken the persistence of gambling behaviors among adolescents (9, 42). Targeting these cognitive biases has shown success in cognitive-behavioral therapies for problem gambling, where individuals learn to recognize the randomness of gambling outcomes and avoid the illusion of control (48–50).

Adolescents' engagement in risk behaviors is also associated with altered regulatory processes, where emotion regulation, temporal discounting, and impulsivity play crucial roles (51–55). In this context, the integration of psychosomatic and neurobiological models has become increasingly relevant in understanding how early experiences and cognitive-emotional frameworks shape decision-making and risk sensitivity. Evidence from mind-body psychotherapy and psychosocial genomics suggests that experiential interventions may promote cognitive restructuring and emotional regulation through bottom-up pathways involving somatic awareness and neuroplastic change (56, 57). The present findings underline the need for prevention efforts specifically tailored to adolescents with high sensation-seeking tendencies. These individuals may benefit from targeted interventions that redirect their need for stimulation toward healthier and structured outlets such as sports, music, or experiential learning activities. School-based programs should incorporate self-regulation training aimed at improving decision-making, emotional control, and delay of gratification. By offering adolescents practical tools to manage impulsivity while maintaining stimulation, such interventions may reduce vulnerability to risky gambling behaviors (58).

These findings carry several implications for prevention efforts aimed at adolescents. First, interventions that strengthen future-oriented thinking, such as goal-setting training or episodic future thinking, may help reduce susceptibility to gambling by fostering long-term planning and self-regulation. Second, programs that challenge gambling-related cognitive distortions (e.g., illusions of control or predictive bias) through psychoeducation and cognitive restructuring could reduce risky beliefs that sustain gambling behavior. Finally, recognizing that adolescents high in sensation-seeking are more vulnerable to gambling, interventions may benefit from incorporating components focused on healthy risk-taking and emotional regulation strategies.

Preventive actions can be effectively implemented in schools or via online platforms, using serious games and interactive activities to engage youth (6, 17). Recent reviews also emphasize the importance of tailoring content to impulsivity profiles and cognitive vulnerabilities in order to improve intervention uptake and efficacy (8, 59). Taken together, our findings underscore the need for multi-component interventions that address both dispositional traits and temporal-cognitive styles.

### Limitations and future research

While this study provides valuable insights into the interplay of temporal perspectives, sensation-seeking, and cognitive distortions in adolescent gambling, several limitations should be acknowledged. First, the cross-sectional design limits causal interpretations of the relationships among these variables. Longitudinal studies could clarify the directionality of these associations, particularly whether present-focused perspectives and sensation-seeking predispose adolescents to gambling or whether gambling itself reinforces these traits (34).

The sample, while diverse, was region-specific to Lazio, Italy, which may limit the generalizability of findings to other cultural contexts. Research in other regions and with culturally diverse populations could validate and extend these results. Lastly, self-report measures, though widely used, are susceptible to social desirability bias. Future studies might incorporate behavioral or observational measures to assess gambling tendencies and sensation-seeking more objectively (42).

While no direct measure of temporal discounting was included in the present study, the use of time perspective scales offers an indirect yet meaningful proxy for adolescents’ orientation toward delayed versus immediate outcomes. Future research may benefit from integrating behavioral discounting tasks to more precisely capture impulsive decision-making mechanisms underlying gambling risk.

Future research may benefit from testing mediation models or full structural equation models to explore indirect pathways between dispositional traits, cognitive factors, and gambling behavior. Such models could clarify whether variables like cognitive distortions mediate the effect of sensation seeking on gambling severity, offering a more nuanced understanding of vulnerability structures.

Although the current study did not test interactions between sensation seeking and cognitive distortions, this combination may represent a particularly potent risk profile for adolescent gambling. Sensation seeking may increase the propensity to engage in novel or risky behaviors, while cognitive distortions may reinforce erroneous beliefs about control or probability in gambling. Future research should examine whether the joint presence of high sensation seeking and strong cognitive distortions amplifies vulnerability to gambling problems, potentially in a synergistic rather than additive fashion.

One key limitation of this study is its cross-sectional design, which precludes causal inferences. Although we found significant associations between temporal perspectives, cognitive distortions, and gambling severity, these findings reflect correlational relationships. It is possible, for instance, that engaging in gambling behaviors reinforces present-oriented thinking or cognitive biases, rather than the reverse. Future research using longitudinal designs is necessary to examine the directionality of these effects and clarify whether temporal perspectives and cognitive distortions serve as precursors to gambling behavior or emerge as consequences of repeated gambling experiences. Prospective studies could help identify critical developmental periods during which these psychological traits exert the greatest influence on gambling onset or escalation.

We acknowledge also the limitations related to the skewed distribution of gambling severity and the modest role of demographic covariates. To mitigate these issues, we employed robust analytic techniques and clearly distinguished exploratory subgroup observations from confirmatory findings. While some discrepancies emerged between correlational and subgroup analyses, we refrain from implying selectivity without formal statistical comparison and caution against overinterpreting isolated effects given reduced statistical power in subgroups. Although some divergences in correlational patterns were observed across subgroups, no formal r-to-z comparisons were conducted (60); therefore, we refrain from inferring statistical selectivity between coefficients.

It is also important to consider the limitations inherent in self-report data, particularly in adolescents. While anonymity may reduce impression management, the absence of a dedicated social desirability scale limits our ability to quantify potential response bias. Future research could benefit from including such measures or adopting implicit or behavioral tools to triangulate self-reported gambling behaviors and cognitive tendencies.

## Conclusion

This study highlights the significant role of temporal perspectives, sensation-seeking, and cognitive distortions in adolescent gambling behaviors. The findings confirm that a present-hedonistic orientation is a strong predictor of both gambling frequency and severity, underscoring the impact of a focus on immediate gratification in risky behaviors. Similarly, present-fatalistic perspectives correlate with gambling severity, suggesting that a fatalistic view of the future may foster escapism through gambling. Conversely, a future-oriented perspective appears to act as a protective factor, as adolescents who prioritize long-term goals exhibit reduced gambling tendencies.

Educational programs aimed at developing future-oriented thinking in adolescents could be beneficial. By encouraging goal-setting and long-term planning, these programs can help young people build the cognitive tools necessary to resist impulsive decisions, including gambling.

Cognitive restructuring interventions can be effective in addressing erroneous beliefs about control and predictability in gambling. By challenging these cognitive biases, adolescents may become more aware of gambling risks and reduce persistent gambling behaviors. Since high sensation-seeking individuals are more prone to gambling, offering structured activities that fulfill thrill-seeking needs, such as sports or adventure programs, can provide safer, controlled outlets for risk-taking.

## Data Availability

The raw data supporting the conclusions of this article will be made available by the authors, without undue reservation.
