# Supplementary material for: Temporal perspectives, sensation-seeking, and cognitive distortions as predictors of adolescent gambling behavior: a study in Italian high schools
Source: Front Psychiatry. 2025 Aug 4;16:1602316. doi: 10.3389/fpsyt.2025.1602316 (PMC12360077; doi:10.3389/fpsyt.2025.1602316)
Supplement: Supplementary file 1 [file DataSheet1.pdf]

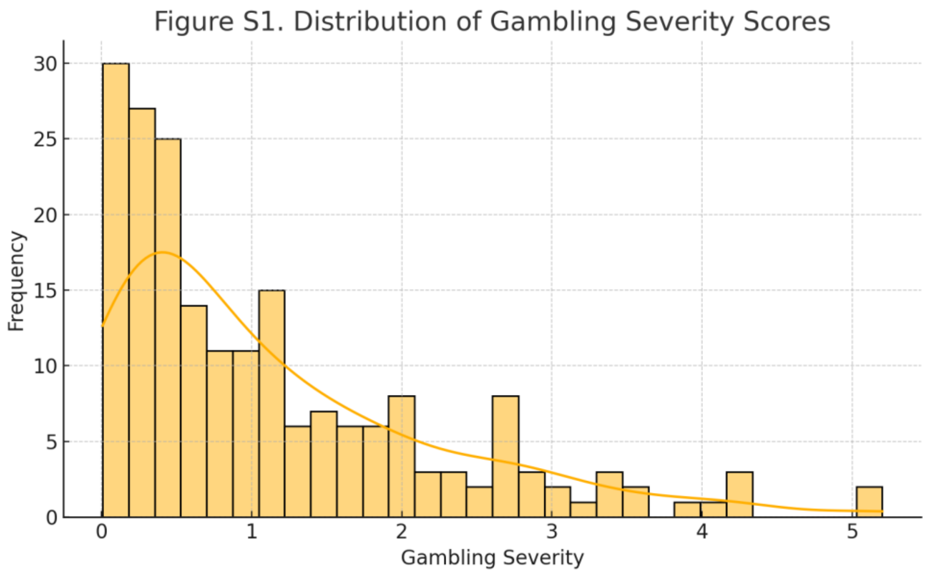

Supplementary Table S2. Zero-order correlations with Gambling Severity	
Variable	r with Gambling Severity
Age	-0.02
Sex (0 = F,1 = M)	0.10
SES	0.02

Supplementary Table S2 Zero-order Pearson correlations between demographic variables and gambling severity (N = 1,424).

Note: Gambling severity shows a small-to-moderate positive correlation with sex (coded as 0 = female, 1 = male), and marginal associations with age and socioeconomic status (SES). These variables were included as covariates in subsequent regression analyses.
